# Congenital hepatic fibrosis and its mimics: a clinicopathologic study of 19 cases at a single institution

**DOI:** 10.1186/s13000-021-01142-y

**Published:** 2021-08-30

**Authors:** Irene Y. Chen, Christa L. Whitney-Miller, Xiaoyan Liao

**Affiliations:** Department of Pathology and Laboratory Medicine, University of Rochester Medical Center, 601 Elmwood Avenue, Box 626, Rochester, NY 14642 USA

**Keywords:** Congenital hepatic fibrosis, Portal hypertension, Hepatoportal sclerosis, Nodular regenerative hyperplasia

## Abstract

**Background:**

Congenital hepatic fibrosis (CHF) is a rare inherited form of ductal plate malformation associated with polycystic kidney disease. The diagnosis requires histopathologic confirmation, but can be challenging to distinguish from other undefined fibrocystic liver diseases. We aimed to describe the clinicopathologic features of congenital hepatic fibrosis (CHF), with comparisons to other entities that may clinically and/or histologically mimic CHF.

**Methods:**

Nineteen cases that carried a clinical and/or histologic impression of CHF were identified at our institution, of which the histology was reassessed and reappraised into two categories: CHF (*n*=13) and mimics (*n*=6). The clinicopathologic features between the two groups were analyzed and compared.

**Results:**

The CHF group was further sub-classified into those with clinical suspicion (CHF-c, *n*=8) and those as incidental histology findings (CHF-i, *n*=5). Patients of CHF-i were much older than CHF-c or mimics (*P*<0.05). Male and female were equally affected. Six of 8 CHF-c (66.7%) had concurrent kidney diseases, including 5 polycystic kidney diseases. Five of 6 mimics (83.3%) had various kidney diseases, including nephronophthisis, Alport syndrome, renal agenesis, and nephrolithiasis. None of the CHF-i patients had kidney disease, but 3 were associated with hepatic carcinomas. Histology analysis demonstrated characteristic triads (bile duct abnormalities, portal vein hypoplasia, and fibrosis) in all CHF cases. One mimic had paucity of intrahepatic bile ducts, while the other 5 mimics showed abnormal portal veins and nodular regenerative hyperplasia consistent with hepatoportal sclerosis (HPS).

**Conclusions:**

Our study demonstrates classic histology triad of CHF despite a wide spectrum of clinical presentations. HPS is unexpectedly a clinical mimicker of CHF, which can be distinguished histologically.

## Background

Congenital hepatic fibrosis (CHF) is a rare inherited form of fibrocystic liver disease caused by incompletely remodeled ductal plates of interlobular bile ducts, resulting in excessive embryologic bile ducts, ectasia, and involution of the ductal plate remnants with fibrosis [1]. This process, referred to as ductal plate malformation (DPM), is an essential precursor lesion of all variants of fibrocystic liver diseases depending on the affected levels of the intrahepatic biliary tree: at the interlobular bile ducts it produces the anatomical-clinical entity CHF, at the larger segmental or septal bile ducts it causes cystic dilatation and is designated as Caroli’s disease, and at the smaller ducts of more terminal portal tract ramifications it results in microharmatoma called von Meyenburg complex (VMC) [2, 3]. Because of the shared pathogenesis between CHF and other forms of DPM, the clinical and pathologic manifestations of CHF are often heterogeneous, rendering difficulties for diagnosis [3]. For example, VMC can be seen in patients with CHF, but localized VMC is often an incidental harmless finding [4, 5]. Caroli’s disease is defined as congenital dilation of the larger intrahepatic bile ducts without further histologic abnormalities. When associated with CHF, the combined form is called Caroli’s syndrome [3, 6]. Both CHF and Caroli’s disease or syndrome are rare, and many pathologists are unfamiliar with those terminologies, leading to confusion and misinterpretations.

CHF is usually diagnosed in early infancy or during childhood, with an estimated incidence rate of 1 in 10,000 to 20,000 live births [7]. The most common clinical presentation is portal hypertension, including splenomegaly and variceal bleeding [8]. Like Caroli’s disease, CHF is associated with congenital renal cystic disease with mutations in genes that encode proteins of primary cilia, collectively referred to as “hepatorenal ciliopathies” [3, 9, 10]. The hepatorenal ciliopathies include autosomal dominant polycystic kidney disease (ADPKD), autosomal recessive polycystic kidney disease (ARPKD), and other rare syndromes such as Joubert syndrome (OMIM #213300), Meckel-Gruber syndrome (OMIM #249000), Bardet-Biedl syndrome (OMIM #209900), and nephronophthisis (OMIN #256100) [2]. CHF may also occur in isolation without manifestation in other organs. The clinical expression is thus highly variable. In severe cases it can be rapidly progressive, while in others it is slow and torpid, or may even spontaneously regress, especially in adult patients [3]. The presentation of portal hypertension in the setting of chronic kidney disease often leads to high suspicion of CHF in pediatric or young adult patients, for whom CHF can be over diagnosed. On the other hand, the diagnosis of CHF in an asymptomatic adult patient without concurrent kidney disease can be easily overlooked due to failure to include CHF in the differential [11].

In this study, we reviewed our experience in diagnosing CHF in the past 15 years at our institution, focusing on pertinent clinical history and histomorphologic characterization, and its distinction from other clinical and/or histologic mimickers. Our aim was to identify specific features that can help aid in the diagnosis of CHF, while reliably distinguishing it from other entities.

## Methods

### Patients

A retrospective review of the University of Rochester Medical Center pathology database was performed for all liver biopsy and resection specimens between the years 2005-2019 that carried the term “congenital hepatic fibrosis” in diagnosis topline or as a major differential diagnosis in comment. A total of 35 cases were initially identified, among which 12 cases that explicitly denied the possibility of CHF in the pathology report were excluded from this study. For the remaining 23 cases, 4 cases were also excluded, including 2 with no slide for review, 1 with inadequate biopsy material (<10 portal tracts), and 1 donor liver biopsy showing CHF/DPM but no clinical data. This study was approved by the Institutional Review Boards of University of Rochester Medical Center (STUDY00003839).

### Histologic analysis

All hematoxylin and eosin (H&E)-stained sections were reviewed for diagnosis confirmation. Histologic features of bile ducts, vascular abnormalities, cholestasis, and inflammation were analyzed. Fibrosis was graded by Ludwig semiquantitative scale of 0 to 4 [12, 13]. Trichrome, Prussian blue, and Periodic acid–Schiff–diastase (PAS-D, PAS diastase) stains were routinely performed for evaluating fibrosis, iron deposit, and intracytoplasmic inclusions, respectively. Reticulin stain was performed for suspected nodular regenerative hyperplasia. Immunohistochemistry studies for evaluation of bile duct structures were performed using antibodies against cytokeratin 7 (CK7, Cat# GA619, Agilent, Santa Clara, CA) or CK19 (Clone RCK108, Agilent, Santa Clara, CA) according to standard protocols.

### Statistical analysis

Demographic data, clinical history, Imaging findings, and liver function tests were obtained from the electronic medical record. Follow-up (measured in months) was defined as the time from initial diagnosis to death or last clinical examination. All statistical analysis was carried out using Statistical Package for the Social Sciences software (SPSS; Build 1.0.0.1327; copyright 2019, IBM). P-values less than 0.05 were considered statistically significant.

## Results

A total of 19 patients (22 liver biopsies and 4 resection specimens) were included. The diagnosis was re-evaluated and reappraised into two categories: CHF (*n*=13) and mimics (n=6). The CHF cohort was further divided into 2 subgroups: those with clinical suspicion of CHF and histologically confirmed so (CHF-c, *n*=8), and those with no clinical suspicion and diagnosis of CHF as incidental histology findings (CHF-i, *n*=5). The main clinicopathologic features of all 19 patients were summarized Table 1.

**Table 1:** Demographic data

Groups		Case #	Final histologic diagnosis	Specimen^a^	Age	Sex	Pertinent disease or congenital disorders	Portal hyertension (Y/N)	Liver imaging	ALT	AST	ALP	TB	Treatment	Follow-up (months)	Outcome
**CHF**	**CHF-c**	1	CHF	Bx (x2)	11	F	ADPKD	Y	Cirrhosis with multiple liver nodules	39	42	104	2.4	Transjugular intrahepatic portosystemic shunt	144	Alive, stable
		2	CHF	Bx (x2)	6	M	ADPKD	Y	Cirrhosis	155	98	136	0.5	Kidney transplant	144	Alive, stable
		3	CHF	Bx	22	M	ARPKD, spina bifida	Y	Cirrhosis	48	57	706	9.2	Kidney transplant (x3) and liver transplant	93	Alive
		4	CHF	Bx	55	F	Chronic renal failure of unknown cause	Y	Cirrhosis with enhancing lesion	37	84	130	0.3	Dialysis, splenorenal shunt	4	Deceased (sepsis, multiorgan failure)
		5	CHF	Bx	1	M	Congenital hypoglycemia, hyperammonemia, seizure, developmental delay	N	Hepatosplenomegaly, mild central intrahepatic duct dilatation	49	45	388	0.5	N/A	14	Alive, stable
		6	CHF	Bx	18	F	ARPKD, Caroli syndrome, alpha1antitrypsin deficiency (PiMS)	Y	Caroli's disease	23	20	44	1.3	N/A	21	Alive
		7	CHF	Bx	11	M	ADPKD, hereditary pancreatitis (PRSS1 mutationN291)	Y	Cirrhosis	25	28	169	<0.2	N/A	17	Alive
		8	CHF	Bx	19	M	Fragile X syndrome, cognitive impairment (autism)	Y	Multiple bridging band of fibrosis, innumerable (>20) macronodule	279	369	351	1.5	N/A	14	Alive
	**CHF-i**	9	CHF and steatohepatitis	Bx	70	F	Diabetes, Anti-mitochondria 1:320, steatohepatitis	N	Cirrhosis	222	132	118	N/A	N/A	72	Alive, stable
		10	CHF	Bx	65	F	Epigastric pain, weight loss	N	Cirrhosis	17	23	80	0.3	N/A	39	Alive, stable
		11	CHF and hepatocellular carcinoma	Explant	56	M	Hepatitis C cirrhosis	Y	Cirrhosis and liver lesion	44	146	73	42.7	Liver transplant	14	Deceased ( metastatic carcinoma )
		12	CHF and hepatocellular carcinoma	Resection	71	M	Alcohol use	Y	Liver lesion	35	28	70	0.9	N/A	1	Alive
		13	CHF and cholangiocarcinoma	Resection	70	F	Crohn's disease, stage IV descending colon cancer	Y	Liver lesion	45	33	72	0.8	N/A	33	Alive
**Mimics**		14	Paucity of intrahepatic bile duct	Bx (x4)	12	M	Nephronophthisis type 11 (Homozygous TMEM67 gene 1843T>C 2012)	Y	Cirrhosis	169	253	1409	1.3	Kidney transplant	127	Alive, pending Liver transplant
		15	HPS/NRH	Bx + explant	16	M	Nephrolithiasis	Y	Cirrhosis	45	18	107	0.5	Liver transplant	192	Alive
		16	HPS	Bx (x3)	22	F	Hepatosplenomegaly and liver failureat 6 month of age	Y	Cirrhosis	38	30	74	2	N/A	38	Alive
		17	HPS/NRH	Bx	44	F	Unilateral renal agenesis, long-term dialysis dependence	Y	Possible cirrhosis	9	18	181	1	N/A	1	Alive
		18	HPS	Bx	3	F	Alport syndrome (Monoallelic POLG1 mutation), failure to thrive	N	Nodular liver surface	28	45	173	0.2	N/A	45	Deceased (Lemierre syndrome)
		19	HPS/NRH	Bx	62	M	Chronic kidney disease, cardiomyopathy and heart failure	N	Cirrhosis	9	19	53	0.7	N/A	15	Deceased (Heart failure)

*Abbreviation*: *CHF* congenital hepatic fibrosis, *CHF-c* congenital hepatic fibrosis clinically suspected, *CHF-i* congenital hepatic fibrosis as incidental findings, *HPS* hepatoportal sclerosis, *NRH* nodular regenrative hyperplasia, *Bx* biopsy, *N/A* not applicable or not known, *ALT* alanine aminotransferase, *AST* aspartate aminotransferase, *ALP* alkaline phosphatase, *TB* total bilirubin

^a^ The number between brackets represent number of biopsies performed

### Demographic data

The CHF cohort comprised 13 patients, 6 females and 7 males, with a median age of 22 (range: 1-71) years. The mimics group comprised 6 patients, 3 females and 3 males, with a median age of 19 (range: 3-62) years. Patients of CHF-i were much older than patients of CHF-c or mimics (median age 70 vs. 15 vs. 19; P<0.05). There was no sex difference among the groups. For both groups, most patients presented with liver cirrhosis on imaging, portal hypertension, or variably elevated liver enzymes (Table 2).

**Table 2:** Histologic comparison between congenital hepatic fibrosis (CHF) and its mimics

Histologic parameters		CHF (*n*=13)	Mimics (*n*=6)	*P*-value
**Biliary changes**	Bile ductular-like proliferation	13	3	**<0.05**
	Dilated duct at the center	12	3	N.S.
	Linear lining of ectatic bile ducts along the limiting plate	12	1	**<0.01**
	Bile ducts crossing limiting plates	9	1	N.S.
	Bile duct fusion and anastomosing	11	2	**<0.05**
	VMC/VMC-like structures	6	0	N.S.
	Cholangitis (lymphocytic or neutrophilic)	10	2	N.S.
	Bile duct loss (% loss)	0	1 (68% loss)	N.S.
**Vascular changes**	Portal vein obliteration/hypoplasia	13	6	N.S.
	Portal vein dilation/herniation	0	6	**<0.001**
	Arterial hyperplasia	13	6	N.S.
	Nodular regenerative hyperplasia	1	4	**<0.05**
**Cholestasis**	Bile plugs in bile ducts and ductules	6	0	N.S.
	Bile plugs in canaliculi (lobular cholestasis)	1	0	N.S.
	Hepatocytic cholestasis	1	0	N.S.
	Bile infarcts	0	0	N.S.
	Cholate stasis	0	0	N.S.
**Inflammation**	Portal inflammation	7	1	N.S.
	Interface hepatitis	0	0	N.S.
	Lymphoid aggregates	2	0	N.S.
**Fibrosis**	Portal tract expansion with fibrosis	13	1	**<0.05**
**Fibrosis stage: Ludwig (0-4)**	Stage 1 (portal fibrosis)	1	1	N.S.
	Stage 2 (periportal fibrosis)	3	3	
	Stage 3 (bridging fibrosis)	6	1	
	Stage 4 (cirrhosis)	3	1	

Chronic kidney disease was noted in 6 of 8 (66.7%) CHF-c patients, 3 ADPKD, 2 ARPKD, and one not further specified. For the other two CHF-c patients without kidney disease, one had fragile X syndrome, and the other one had congenital hyperammonemia, jaundice, and seizures of unknown reasons. Interestingly, 3 patients who had ADPKD or ARPKD also had other congenital disorders such as spina bifida, hereditary pancreatitis, and alpha-1-antitrypsin deficiency. In contrast, none of the CHF-i patients had concurrent kidney disease or congenital disorders, and the diagnoses of CHF were incidental histologic findings for other purposes: biopsies to rule out primary biliary cholangitis (*n*=2), and resections for either hepatocellular carcinomas (*n*=2), or metastatic colon cancer with incidental finding of cholangiocarcinoma (*n*=1).

In the mimics group, 5 of 6 (83.3%) patients had kidney diseases, including nephronophthisis type 11 with homozygous *TMEM67* gene mutation at 1843T>C 2012, Alport syndrome with monoalleic *POLG1* mutation, unilateral renal agenesis, nephrolithiasis, and nonspecific chronic cardiac and renal failure. In the settings of kidney disease and portal hypertension, a clinical suspicion of CHF was raised. One mimic did not have concurrent kidney disease, but was clinically diagnosed as CHF given hepatosplenomegaly and liver failure at 6 months of age (case 16).

### Histologic features

**Fig. 1:**
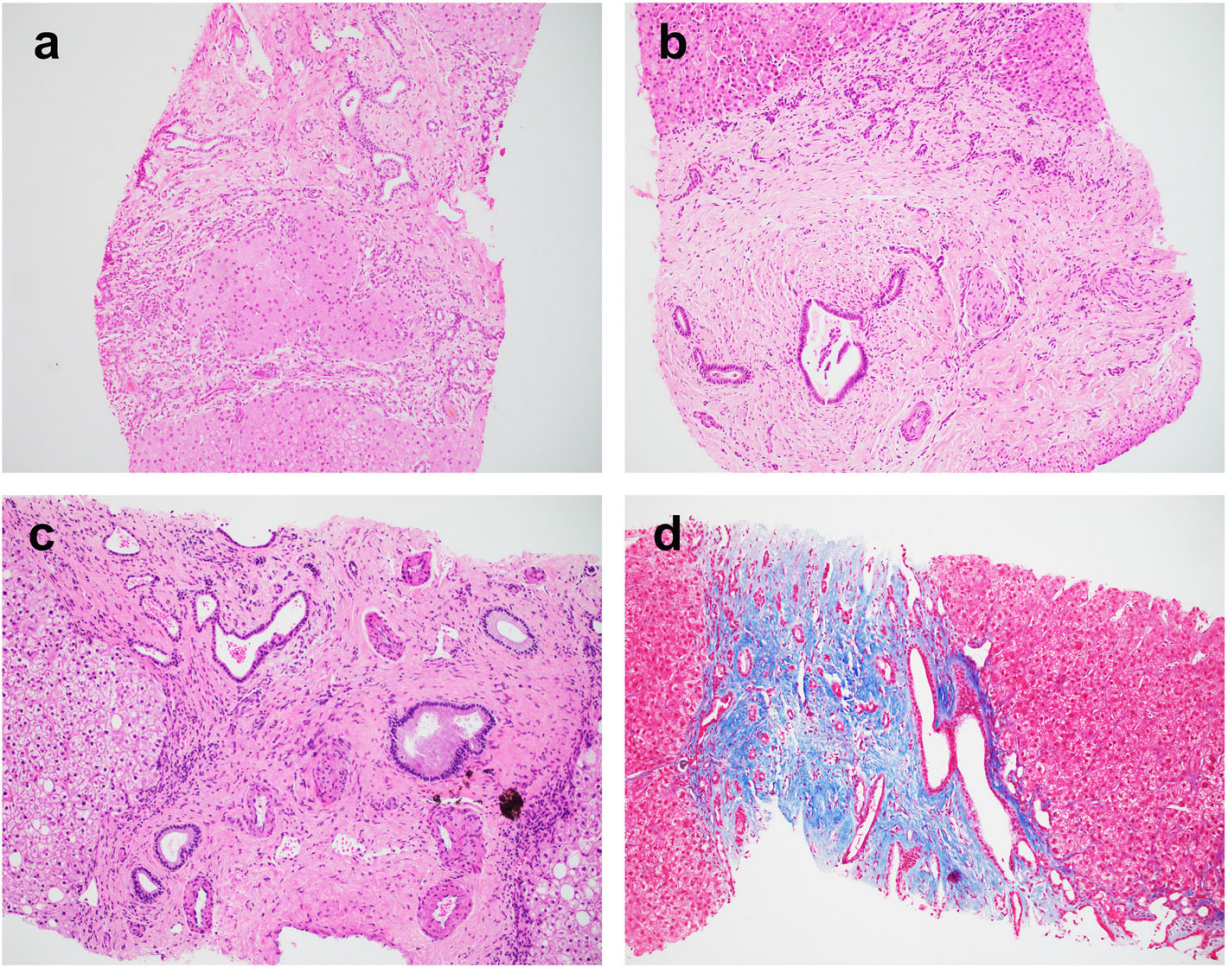
Representative histology of congenital hepatic fibrosis. **a** Case 1. Broad fibrous bands containing abnormal bile duct profiles with anastomosing and ectasia separate the parenchyma into nodules without hepatocyte regeneration. **b** Case 3. The portal tract is expanded with prominent fibrosis. There are centrally located bile ducts with luminal dilatation and numerous smaller bile ducts at the limiting plate. The portal vein is hardly appreciated. **c** Case 9. The bile ducts are ectatic and irregular. The portal veins are small and the portal arteries are prominent and supernumerous. **d** Case 4. Trichrome stain highlights the portal fibrosis with embedded abnormal bile ducts. Magnification: 100x

**Fig. 2:**
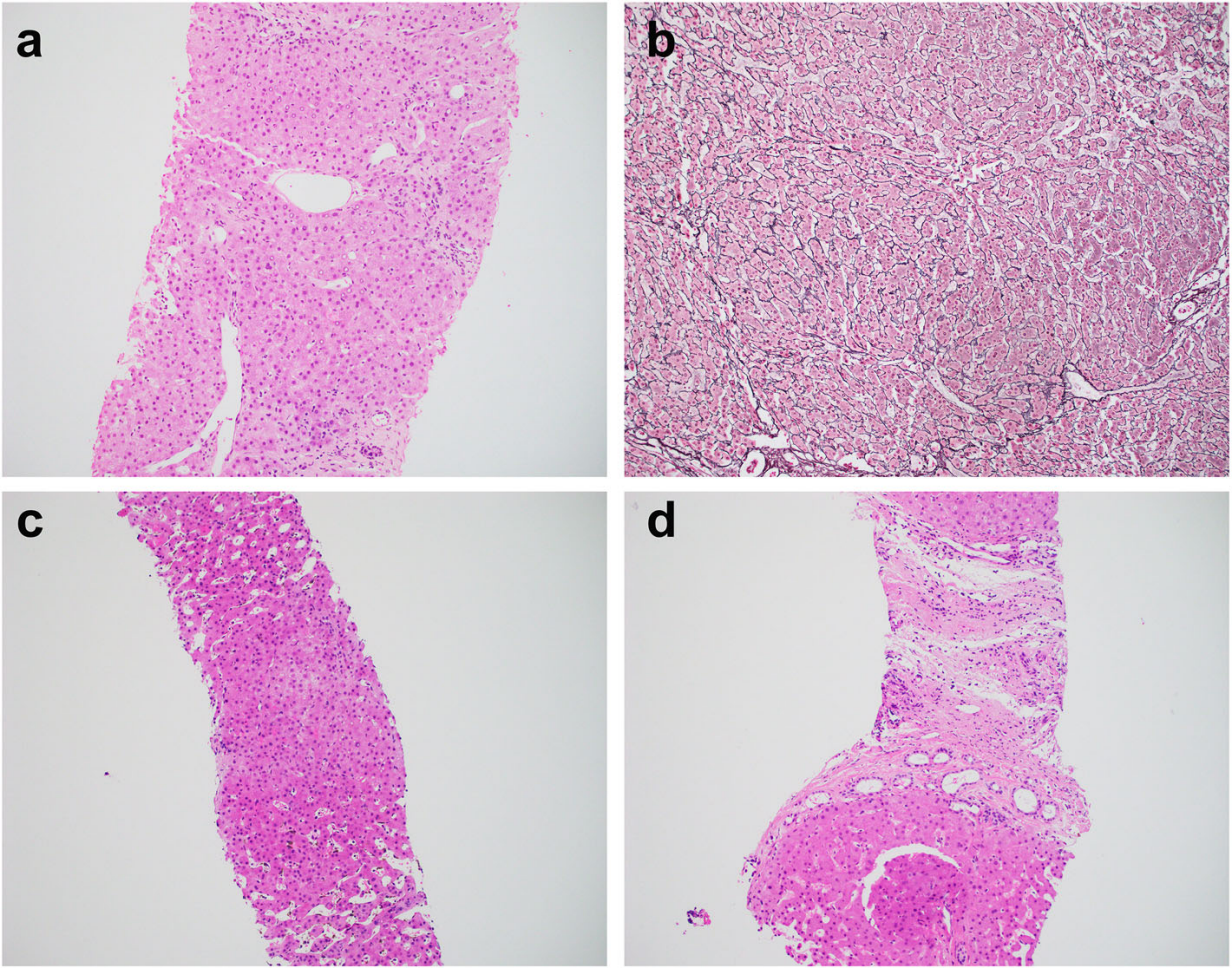
Mimics of CHF with histologic features of hepatoportal sclerosis. **a-b** Case 15. Biopsy (**a**) showing portal vein abnormalities with alternating obliteration/hypoplasia in some portal tracts, but dilation and herniation in other portal tracts. Reticulin stain in explant liver (**b**) confirms nodular regenerative hyperplasia. **c** Case 17. Sinusoidal dilatation with Kupffer cell hyperplasia and iron deposit. **d** Case 19. Focal and mild bile duct abnormalities that mimics ductal plate malformation. Magnification: 100x

**Fig. 3:**
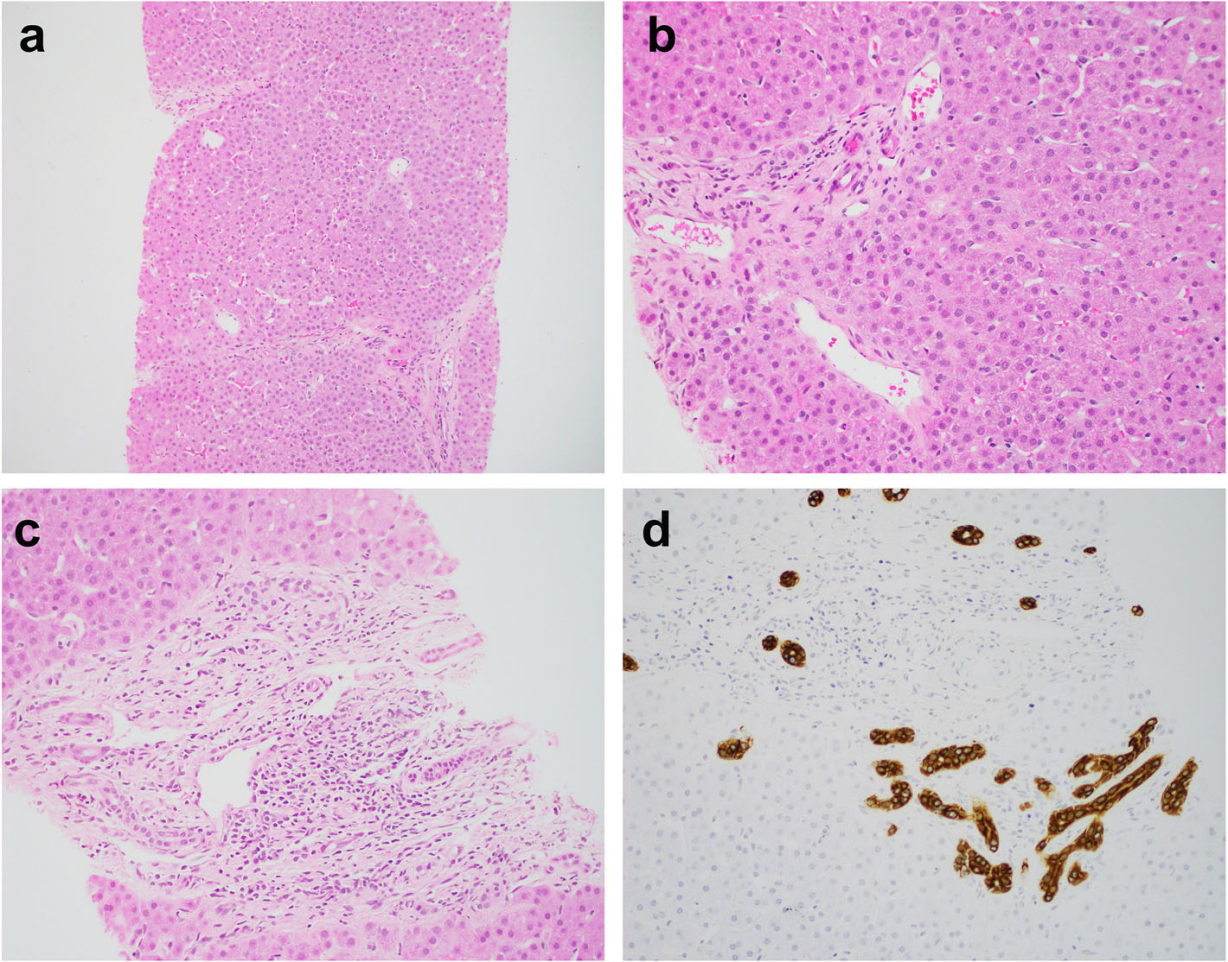
Paucity of intrahepatic bile ducts in a patient with nephronophthisis (case 14). **a-b** The liver shows thin delicate portal/periportal fibrosis and eccentrically located portal veins with dilatation and herniation. Bile duct is missing in approximately 68% portal tracts. **c-d** In one portal tract there are residual bile ducts highlighted by CK19 immunohistochemistry, somewhat resembling ductal plate malformation. Magnification: **a-b**: 100X, **c-d**: 200x

The histopathology comparisons between the CHF and mimics are summarized in Table 2. All CHF cases demonstrated classic histology triads: (1) abnormal bile duct profiles; (2) hypoplastic portal vein branches, and (3) progressive fibrosis (Fig. 1). One of the mimics demonstrated paucity of intrahepatic bile duct, while the other 5 mimics unexpectedly showed features consistent with hepatoportal sclerosis (HPS, Figs. 2 and 3).

#### Biliary changes

The most characteristic feature of the bile duct abnormalities in CHF was numerous small ectatic bile ducts lining along the limiting plate and at the center of the portal tracts, some crossing the hepatic parenchyma. Some ducts contained inspissated bile, resembling VMC, or were fused and anastomosing, with associated neutrophils and lymphocytes, resembling bile ductular reaction (Fig. 1). VMC-like structures or VMC were noted in 3 of 10 biopsies and all 3 resections in CHF cases. Cholangitis, defined as lymphocytic or neutrophilic infiltrates of the bile duct wall or epithelium, was present in majority of the CHF cases. In contrast, the bile duct abnormalities in the mimics were mostly focal or mild, although occasional bile duct ectasia or linear lining along the limiting plate was present (Fig. 2). Bile duct damage or duct loss was rare in both groups. However, one mimic demonstrated ductopenia (68% loss) in two consecutive liver biopsies, with a rare portal tract showing focal residual embryologic type bile ducts at the limiting plate resembling DPM (Fig. 3).

#### Vascular changes

The portal veins in CHF were obliterated, rudimentary or hypoplastic. Herniation and dilatation were very rare compared to the mimics group (P<0.05). The arteries were often thickened, sometimes supernumerous, coursing through the fibrous stroma, which were seen in both CHF and mimics. Indeed, the hallmark feature of HPS was alternating portal vein obliteration/hypoplasia with herniation/dilation, resulting in nodular regenerative hyperplasia (Fig. 2), which was noted at least focally in 4 of 6 mimics, but rarely in CHF (*P*<0.05).

#### Cholestasis and portal inflammation

In CHF, the dilated bile ducts sometimes contained inspissated bile, suggesting communication with the main biliary tree. Cholestasis or cholate stasis was not a prominent feature in either CHF or mimics. Both CHF-c and mimics had no significant portal or lobular inflammation unless superimposed with other pathologic findings such as cancer in CHF-i group.

#### Fibrosis

Fibrosis was evaluated in conjunction with trichrome stains. Portal tract expansion with prominent fibrosis distending the portal tract contour was an invariable feature in CHF (Fig. 1). When fibrosis advanced into cirrhosis, the fibrous band contains numerous small ectatic bile ducts. In contrast, the mimics more likely exhibited slender and hypocellular fibrosis, including the one with paucity of bile ducts. Despite imaging showing cirrhosis in most cases, only half of the CHF patients demonstrated bridging fibrosis (stage 3), while half of the mimics showed periportal fibrosis (stage 2).

#### Malignancies

**Fig. 4:**
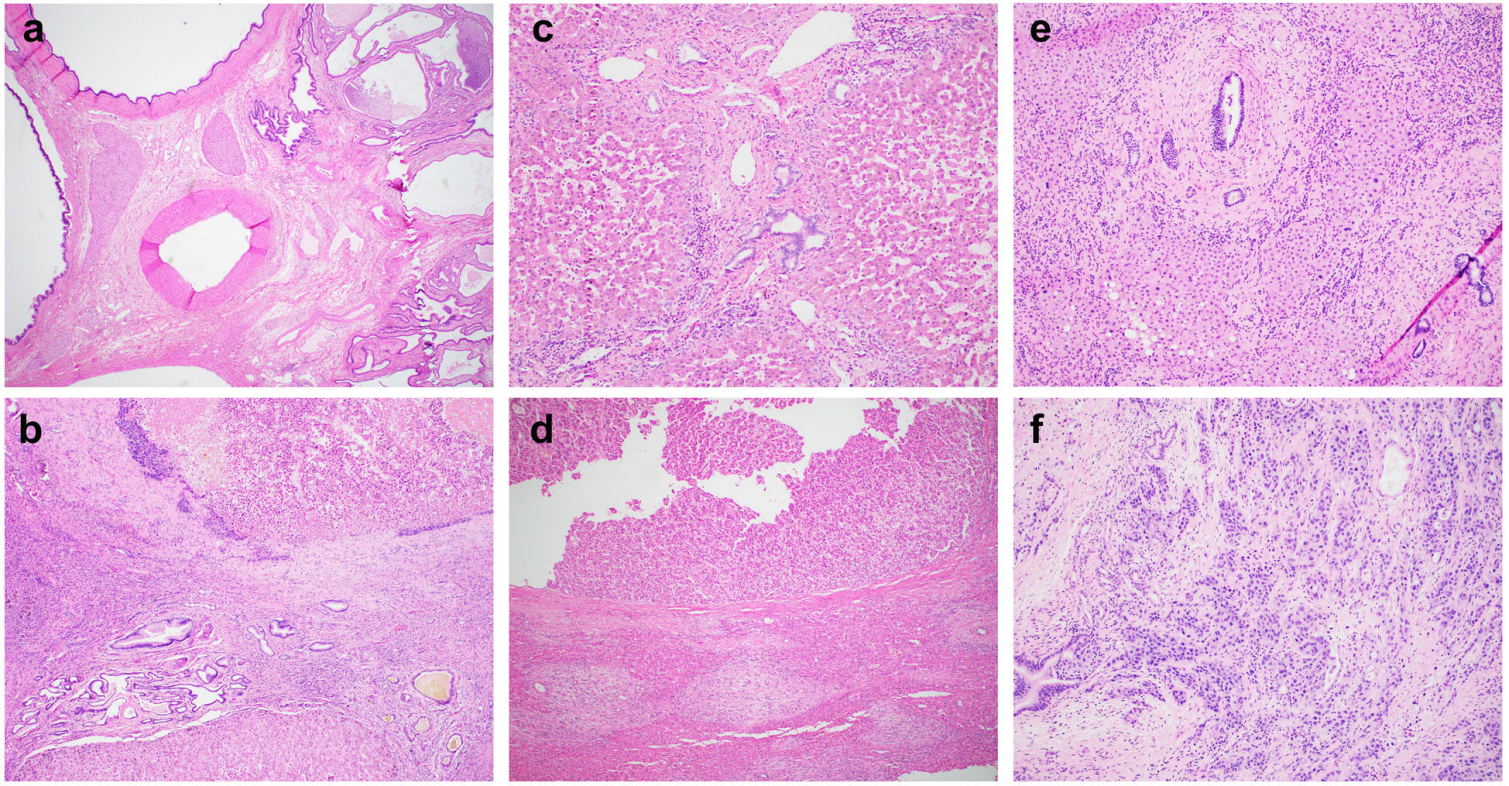
Carcinomas associated with congenital hepatic fibrosis. Case 11 (**a-b**) showing numerous cystically dilated bile ducts in hilar area (**a**) and hepatocellular carcinoma (**b**, upper half) with adjacent portal tracts (**b**, lower half). Case 12 (**c-d**) showing background congenital hepatic fibrosis (**c**) and hepatocellular carcinoma (**d**. upper half) with adjacent portal tracts (**d**, lower half). Case 13 (**e-f**) showing background congenital hepatic fibrosis (**e**) and incidental findings of cholangiocarcinoma (**f**) involving a benign dilated duct. Magnification: **a**: 20X; **b, c, e**, **f**: 100X; **d**: 40X

Three CHF-i cases were associated with primary hepatic malignancies (Fig. 4). The two cases of hepatocellular carcinoma each had additional risk factors (hepatitis C and steatohepatitis), with CHF being incidental histologic findings. The cholangiocarcinoma case was even more dramatic: the patient had a history of Crohn’s disease and stage IV colon adenocarcinoma with liver metastasis. On resection, there were numerous cystically dilated bile ducts at hilar parenchyma and a small focus of cholangiocarcinoma associated with background CHF suggesting undiagnosed Caroli’s syndrome. In all three cases, the background DPM/CHF was present both away from the tumor (Fig. 4a, c, e) and adjacent to the tumor (Fig. 4b, d, f), which was different than nonspecific mass effect (portal edema, inflammation, and bile ductular reaction, etc).

### Clinical outcomes

The treatment strategy for both CHF and mimics were similar. The main goal was to manage comorbidities especially kidney disease (dialysis, kidney transplant), and symptoms of portal hypertension (shunting, liver transplant). After a median follow-up of 33 (range: 1-192) months, four patients, including 2 CHF and 2 mimics, died of diseases. Kaplan-Meier survival analysis revealed no significant difference between these two groups (data not shown).

## Discussion

Bile duct embryogenesis is a complicated process that involves many genetic elements and signaling pathways [14]. CHF is commonly viewed as a developmental disorder in pediatric patients rather than an adult liver disease [15]. Echoing the recent case series of 5 adult CHF [11]. we broadened our research not only to analyze all pediatric and adult CHF cases, but also cases that mimicked CHF clinically and/or histologically. Strikingly, some of the mimics were still labeled as CHF in the clinical notes, partially because the pathologist who signed out the report did not completely exclude the possibility of CHF, leading to diagnostic confusion. Those cases were thus analyzed as a control group for comparison to CHF cases, to clarify the fundamental concepts of DPM/CHF for both clinician and pathologist colleagues.

In our study cohort, most of the pediatric CHF cases had either ADPKD or ARPKD, substantiating the concept of “hepatorenal ciliopathies” where mutations in genes that encode the proteins of primary cilia are the underlying causes for the histologic abnormalities [10, 16]. Interestingly, they often also had other congenital issues, such as spina bifida, hereditary pancreatitis, alpha-1-antitrypsin deficiency, or even Fragile X syndrome. There are currently no reported genetic associations between CHF and the above-mentioned anomalies, and therefore the findings may represent coincidences rather than true mechanistic relationships. Nevertheless, the various congenital disorders in the context of liver dysfunction did increase the awareness of possible CHF, leading to liver biopsies for histologic confirmation.

It may also be the high frequency of kidney disease and relatively young age that have led to biased impression in the patient group of “mimics”, who often had elevated liver enzymes and/or symptoms of portal hypertensions that masquerade as CHF. Histologically, most of those cases had portal fibrosis and vascular abnormalities that would be best classified as HPS, an enigmatic disease entity that is thought to be idiopathic, but frequently associated with many nonspecific disease conditions, such infection, autoimmune disorders, inflammatory conditions, heart or kidney diseases, portal vein thrombosis, etc. [2, 17, 18]. HPS occurs in both children and adults. It is one of the causes for noncirrhotic portal hypertension. In severe cases, liver transplant is required, such as our case 15. The mechanisms of developing HPS and liver dysfunction in a patient with chronic kidney disease is not clear, but may be related to alteration of volume distribution in the portal vein branches, or excessive toxin (such as uremia) compromising endothelial functions. The bile duct abnormalities in those patients, however, are often focal and mild, distinct from DPM/CHF.

While most mimics showed features of HPS, there is one exception, the nephronophthisis patient (case 14). Ironically, nephronophthisis as a rare autosomal recessive kidney disease has been long recognized as a member of the “hepatorenal ciliopathies”. The association between nephronophthisis and CHF was first established by a study in 1973 [19], and subsequently described in multiple case reports [20, 21]. Nonetheless, it is unclear if the liver manifestation in those nephronophthisis patients are truly DPM/CHF or just hepatic fibrosis [22]. In fact, Talia reported the first case of paucity of intrahepatic bile duct in a nephronophthisis patient in 1987 [23], features very similar to our case. Although “CHF” is still frequently used in textbooks as one of the complications in nephronophthisis, the pathogenesis process may be not be the same as CHF, but rather a variant of biliary atresia or Alagille’s syndrome which has been described in scarce case reports [24, 25].

Fortunately, despite the wide variations in clinical presentations, the liver biopsy in CHF patients often shows classic histomorphology for which we coined the term “triads” to describe the abnormal bile duct profile, hypoplastic portal vein, and progressive fibrosis that are almost invariably presented in every case [3]. The molecular mechanisms of this “vein-duct” interaction was nicely illustrated in a *Anks6* knock-out mouse study demonstrating the involvement of Hippo-YAP/TAZ angiogenesis pathway during bile duct embryogenesis [26], suggesting a finely tuned interaction among the portal veins, ductal plates, and the intervening mesenchyme during ductal plate remodeling [24]. DPM is also a known risk factor for hepatobiliary neoplasms, more frequently cholangiocarcinoma than hepatocellular carcinoma [27–29]. Interestingly, at least 2 of the three carcinoma cases in our study cohort also had other risk factors for carcinogenesis, rendering the CHF as a background disease, which may act as a synergistic factor rather than direct cause of neoplasia. Nevertheless, keeping in mind that CHF is associated with risk of primary liver malignancies makes the early diagnosis of CHF/DPM necessary for proper patient management and follow-up.

In summary, our study affirms classic histology triads as the characteristic features of CHF despite wide spectrum of clinical presentations. We recommend to topline the diagnosis of DPM/CHF in pathology report if histology fits regardless of clinical pictures. CHF is associated with hepatic neoplasms, which may serve as precursor lesion of cholangiocarcinoma, or synergize with other risk factors for hepatocellular carcinoma. HPS is unexpectedly a close mimicker of CHF; the alternating dilation and occlusion of portal veins and nodular regenerative hyperplasia are distinct features to separate from CHF. Our interesting case of intrahepatic paucity of bile ducts associated with nephronophthisis challenges the established association between these two disease entities, the exact pathogenesis of which warrants further studies.

## Data Availability

The datasets generated and/or analyzed in this study are available from the corresponding author upon reasonable request.

## Abbreviations

CHF: Congenital hepatic fibrosis
DPM: Ductal plate malformation
HPS: Hepatoportal sclerosis
NRH: Nodular regenerative hyperplasia

## References

[1] Johnson CA, Gissen P, Sergi C (2003). Molecular pathology and genetics of congenital hepatorenal fibrocystic syndromes. J Med Genet.

[2] Saxena R. Practical hepatic pathology: A diagnostic approach. 2nd Ed. Philadelphia: Elsevier; 2018.

[3] Desmet VJ (1992). Congenital diseases of intrahepatic bile ducts: variations on the theme "ductal plate malformation". Hepatology.

[4] Mimatsu K (2008). Preoperatively undetected solitary bile duct hamartoma (von Meyenburg complex) associated with esophageal carcinoma. Int J Clin Oncol.

[5] Redston MS, Wanless IR (1996). The hepatic von Meyenburg complex: prevalence and association with hepatic and renal cysts among 2843 autopsies [corrected]. Mod Pathol.

[6] Acevedo E (2020). Caroli's Syndrome: An Early Presentation. Cureus.

[7] Parkash A (2016). Congenital hepatic fibrosis: clinical presentation, laboratory features and management at a tertiary care hospital of Lahore. J Pak Med Assoc.

[8] Shorbagi A, Bayraktar Y (2010). Experience of a single center with congenital hepatic fibrosis: a review of the literature. World J Gastroenterol.

[9] Bergmann C (2017). Genetics of Autosomal Recessive Polycystic Kidney Disease and Its Differential Diagnoses. Front Pediatr.

[10] Gunay-Aygun M (2009). Liver and kidney disease in ciliopathies. Am J Med Genet C Semin Med Genet.

[11] Alsomali MI (2020). Diagnosis of Congenital Hepatic Fibrosis in Adulthood. Am J Clin Pathol.

[12] Ludwig J, Dickson ER, McDonald GS (1978). Staging of chronic nonsuppurative destructive cholangitis (syndrome of primary biliary cirrhosis). Virchows Arch A Pathol Anat Histol.

[13] Ishak K (1995). Histological grading and staging of chronic hepatitis. J Hepatol.

[14] Lemaigre FP (2003). Development of the biliary tract. Mech Dev.

[15] Vajro P (2014). Management of adults with paediatric-onset chronic liver disease: strategic issues for transition care. Dig Liver Dis.

[16] The polycystic kidney disease 1 gene encodes a 14 kb transcript and lies within a duplicated region on chromosome 16. The European Polycystic Kidney Disease Consortium. Cell. 1994;77(6):881–94. 10.1016/0092-8674(94)90137-6 8004675

[17] Fiel MI, Schiano TD (2019). Idiopathic noncirrhotic portal hypertension. Semin Diagn Pathol.

[18] Riggio O (2016). Idiopathic noncirrhotic portal hypertension: current perspectives. Hepat Med.

[19] Boichis H (1973). Congenital hepatic fibrosis and nephronophthisis. A family study. Q J Med.

[20] Witzleben CL, Sharp AR (1982). "Nephronophthisis-congenital hepatic fibrosis": an additional hepatorenal disorder. Hum Pathol.

[21] Gomez Campdera FJ (1981). Nephronophthisis: study of 10 cases. Incidence, natural history and associated pathology (author's transl). Med Clin (Barc).

[22] Hildebrandt F, Zhou W (2007). Nephronophthisis-associated ciliopathies. J Am Soc Nephrol.

[23] Tolia V (1987). Renal abnormalities in paucity of interlobular bile ducts. J Pediatr Gastroenterol Nutr.

[24] Emerick KM (1999). Features of Alagille syndrome in 92 patients: frequency and relation to prognosis. Hepatology.

[25] Martin SR, Garel L, Alvarez F (1996). Alagille's syndrome associated with cystic renal disease. Arch Dis Child.

[26] Airik M (2020). Loss of Anks6 leads to YAP deficiency and liver abnormalities. Hum Mol Genet.

[27] Srinath A, Shneider BL (2012). Congenital hepatic fibrosis and autosomal recessive polycystic kidney disease. J Pediatr Gastroenterol Nutr.

[28] Yonem O (2006). Is congenital hepatic fibrosis a pure liver disease?. Am J Gastroenterol.

[29] Choe JY, Kim H (2014). Intrahepatic cholangiocarcinoma with predominant ductal plate malformation pattern. Clin Mol Hepatol.

